# Study protocol—Evoked craving in high-dose benzodiazepine users

**DOI:** 10.3389/fpsyt.2022.956892

**Published:** 2022-10-13

**Authors:** Lorenzo Zamboni, Silvia Toldo, Francesca Fusina, Matteo Mattiello, Vanessa Mannari, Simone Campagnari, Valentina Schiavone, Alessio Congiu, Giuseppe Verlato, Cristiano Chiamulera, Fabio Lugoboni

**Affiliations:** ^1^Unit of Addiction Medicine, Department of Internal Medicine, Integrated University Hospital of Verona, Policlinico “G.B. Rossi”, Verona, Italy; ^2^Department of Neurosciences, University of Verona, Verona, Italy; ^3^Department of Diagnostics and Public Health, University of Verona, Verona, Italy; ^4^Padova Neuroscience Center, University of Padua, Padua, Italy; ^5^Department of General Psychology, University of Padua, Padua, Italy; ^6^Diagnostics and Public Health-Unit of Epidemiology and Medical Statistics, University of Verona, Verona, Italy

**Keywords:** cue reactivity, benzodiazepine, addiction, virtual reality, abuse

## Abstract

Benzodiazepine (BDZ) abuse, especially concerning high doses of BDZs, is an impairing substance use disorder (SUD) that is often difficult to treat. Craving and cue reactivity (CR) are two important phenomena that have a prominent role in maintaining addiction and triggering relapses in BDZ abuse; nevertheless, they have rarely been addressed in scientific literature. The present study aims to fill these gaps by implementing a highly innovative virtual reality (VR) design to assess the impact of substance-related environmental cues on BDZ craving, as well as their influence on patients’ affective states. Therefore, on one hand, this research will contribute to the assessment of VR feasibility in the study of these phenomena, and, on the other, it will help disentangle the role that CR and craving have on mood and attention, which are equally important factors to consider when treating SUDs. We will recruit a healthy control group and a patient group comprising people seeking treatment for BDZ detoxification. The experimental design will consist of the presentation of three VR scenarios, one neutral, one BDZ-related but without BDZ cues, and another with BDZ cues. The craving will be measured through a virtual analog scale (VAS); the Profile of Mood States (POMS) and Alcohol Attention Scale (AAS) questionnaires in a modified version will also be administered. We will additionally control for VR-induced feelings of sickness by administering the Simulator Sickness Questionnaire (SSQ), and the Presence Questionnaire (PQ) will be used to investigate participants’ sense of presence in virtual environments. We expect patients to exhibit higher levels of craving, and that the craving will be higher after exposure to a cue-related virtual environment as compared to a neutral scenario.

## Introduction

Substance use disorders (SUDs) feature craving as one of their most prominent mechanisms and diagnostic criteria (1). Indeed, craving is involved in the long-term maintenance of abstinence in SUDs, as well as having an important impact on the development of the disorder itself and on the course of the treatment (2–4). Craving is defined as an abrupt urge to consume the target substance (5–7), which often escalates into compulsively seeking the substance and other behaviors related to substance use (8, 9).

Benzodiazepines (BDZs) are positive allosteric modulators of the GABA-A (Gamma-Aminobutyric Acid Type A) receptor (10) which are widely prescribed to treat insomnia and anxiety. Despite their widespread use, studies have shown that BDZs should only be employed in specific clinical situations and preferably for short-term use (11–13). Adverse effects and dependence are associated with their long-term use and should be implemented with extreme caution. Clinicians should also consider short or intermittent treatments, which could have important benefits for patients (14).

About 6–76% of total BDZ users are long-term users. Of these, 15–44% present moderate-to-severe withdrawal symptoms, and 3–4% exhibit dependence (15).

High-dose (HD) BDZ dependence is considered a specific SUD (16), and it consistently reduces the quality of life (17, 18). A cross-sectional survey in France, Germany, Italy, and the UK showed that an estimated 0.14% of the general population took higher-than-recommended doses of anxiolytic medications, while 0.06% reportedly abused hypnotics (19). These data are consistent with those reported by a study conducted in Switzerland, which revealed an incidence rate of 0.16% concerning high-dose BDZ use (20) and points toward HD BDZ abusers being around 1.5 million in Europe and 600,000 in the United States.

Long-term BDZ use is particularly problematic because it has been found to be associated with anomalies in cognitive functions such as attention, memory, and learning. It also exposes patients to a higher risk of delirium, cognitive decline, and accidents (21–30).

To alleviate BDZ withdrawal symptoms, which are particularly impairing for patients, gradual tapering of the dosage or substituting the target BDZ with an equivalent dose of another long-acting benzodiazepine and then tapering are the preferred courses of treatment (31, 32).

Furthermore, BDZs are reportedly secondary drugs of abuse for most individuals, with much fewer patients reporting BDZs as primary drugs of abuse. BDZ abuse is mainly associated with the concurrent abuse of opioids (54.2%) and alcohol (24.7%). Jones et al. (33), in their recent review, reported that about one in five people who abuse alcohol are also benzodiazepine abusers.

Cue reactivity (CR) is a hypersensitivity to motivational stimuli and situations (34). It is considered an adaptive response to salient information (cues) that are present in the environment and it can be evaluated by relying on psychological measures (changes in mood and craving ratings), physiological measures (skin conductance and heart rate), and behavioral measures (gestures/actions) (35). CR is particularly relevant in SUDs, in which it increases craving and facilitates relapses: subjects with a history of substance abuse are particularly sensitive to stimuli and situations which have been previously associated with pleasurable substance effects (36). In this respect, CR is an evolutionary response that may be both a risk factor, when cues are present, and a protective one, when cues are absent: for instance, households with no smoking-related cues have been demonstrated to reduce relapses in smokers (37). Likewise, an external environment may present both protective and precipitating elements. In this perspective, studying the characteristics of various contexts and their function as either risk or protective factors is central in treating and preventing abuse-related behaviors by designing motivationally healthy environments (38). Even though the effects of spatial features on affective states and perception have been extensively studied (39), the role of domestic and urban settings in inducing motivated behaviors is still a largely unexplored topic.

Concerning potential research methods, virtual reality (VR) seems a promising technology to implement in CR paradigms (2, 40, 41). VR consists in the simulation of real-life contexts and environments, which are presented in 3D and are multisensory, comprising auditory, olfactory, visual, and/or tactile inputs (42). Such an approach, being more similar to reality, enhances participants’ *sense of presence*, that is a state of mind in which virtual environments are perceived as similar to real-world ones, and may therefore be more valid than traditional CR paradigms (e.g., 2D screens, photos, etc.) (43–45).

Higher efficacy may be achieved using technical VR features such as immersion within the VR environment and allowing subjects to actively interact with the system through real-time feedback (46). Other important aspects are the inclusion of substance-related stimuli and the presentation of highly realistic environments (47–49).

To the best of our knowledge, there is currently no literature regarding CR and VR in BDZ abuse. Some studies have addressed CR and alcohol abuse (47, 50, 51) and have highlighted the influence that environmental settings have on craving in alcoholics. This work has been inspired by the study by Ryan et al. (50), especially given the scientific rigor they adopted in their research.

## Objectives of the study

### General objective

The general objective of the study is the implementation of a VR protocol to identify the causal relationship between environmental features of a specific setting and craving responses in BDZ abusers.

### Specific objectives

The primary objective of the study is to identify the causal relationship between exposure to environmental cues related to BDZ use and the degree of BDZ craving in abusers.

### Secondary objectives

1. Correlation between the degree of BDZ craving in the various scenarios and measures of mood, affect, attention, sense of presence, and VR malaise in subjects who abuse BDZs.
2. Evaluation of the effectiveness that the three different VR environments have in discriminating between BDZ abusers and control subjects by comparing BDZ craving degree and measures of mood, affect, attention, sense of presence, and VR malaise in the control group vs. those in the experimental group.

## Materials and methods

### Study design

This research will be an experimental study aiming to measure the degree of BDZ craving induced by VR exposure to environments associated with BDZ use (cues) after immersion in a VR scenario of a bedroom only, and then a bedroom in which BDZ bottles will be present. Every subject will be sequentially exposed to both environments to avoid carry-over effects.

There will be two cohorts of participants. The first group will be the control group and it will comprise subjects that do not suffer from SUD. Participants will be recruited from University students, collaborators, and staff of the University or Hospital. The second group will be the experimental one and will be recruited among BDZ-abusing patients seeking treatment at the Department of Addiction Medicine (Department of Internal Medicine, Integrated University Hospital of Verona) due to their inability to autonomously quit using BDZs. All subjects will be informed regarding the procedures and risks associated with the protocol and experimental design and will be asked to sign an informed consent form before participating in the experiment. Before the experimental session starts, we will collect demographic data and administer a series of questionnaires.

The study will consist of a single session lasting about 45 min. Participants will fill out the Profile of Mood States (POMS) questionnaire before and after the session as a pre-VR baseline measure concerning their mood and affective state. After a 3-min VR baseline, we will administer three scenarios, each lasting 3 min. After the baseline and each of the scenarios, subjects will be required to fill out the VAS to report cravings and a modified version of the Alcohol Attention Scale (AAS) questionnaire. At the end of the experimental session, in addition to the POMS, subjects will also be asked to fill in the Presence Questionnaire (PQ) to assess their sense of presence and the Simulator Sickness Questionnaire (SSQ) to assess the presence of possible adverse effects due to VR exposure.

### Materials

#### Virtual reality instrumentation

HTC-VIVE, which is a VR helmet that facilitates feeling immersed in the proposed virtual scenarios and headphones, enhance auditory immersion as well.

This device allows seeing a virtual world with an optical visor which, thanks to new “room scale” technology, transforms the environment into a 3D space in which the user can freely move. This technology, associated with precise head tracking and controls that simulate hand movements, transforms VR into a particularly immersive experience.

The development platform Unity allows to design and build highly immersive VR scenarios that are compatible with HTC-VIVE.

### Procedure

Each subject will be asked to sit in the VR station and will be given all the necessary information regarding the experiment. After signing the informed consent, the subject will give demographic data and will fill out the questionnaires. Before the VR session begins, the participant will be administered the POMS and the VAS on craving. The experimenter will then instruct the participant on how to move in the virtual environment and how to use the HTC-VIVE VR device. The first scenario will be a 3-min baseline simulation during which the subject will familiarize themselves with VR, learn the controls to move around the virtual environment, and practice with the device. In a fixed sequence, the other three scenarios will be shown: house entrance (neutral), bedroom without BDZs, and bedroom with a medicine bottle similar to commercially available BDZ bottles. The subjects will not have to undertake a specific task but will be allowed to freely explore the environment by using the HTC-VIVE PRO Full Kit directional joystick and by moving their head. At the end of each scenario, the subject will remove the visor and headphones and fill out the VAS to report BDZ craving and the modified AAS scale.

Every scenario, including the baseline, will last 3 min. At the end of the last one, the subject will be administered the VAS craving scale, the POMS, the modified AAS, the SSQ, and the PQ.

#### Questionnaires

Anamnesis schedule (Supplementary Appendix 1) with 10 questions.

VAS scale (Supplementary Appendix 2) with a question relative to BDZ craving. The instrument is a single-item visual analog scale with a score ranging from 0 (absent) to 9 (extreme).

The POMS (52) (Supplementary Appendix 3) is widely used to assess mood and to identify possibly problematic affective states. It is a self-report questionnaire, and it is mainly used in clinical psychology, psychotherapy, and medicine. It comprises 58 adjectives that define six mood states: tension-anxiety (T), which describes an overt or covert increase in somatic tension; depression (D), which indicates a depressed mood accompanied by a sense of inadequacy, hopelessness, emotional isolation, melancholy, and guilt; aggression-anger (A), which describes anger and dislike toward others; vigor-activity (V), comprising adjectives that suggest exuberance, energy, euphoria, and optimism; tiredness-indolence (TI), which represents boredom, low energy, and physical fatigue; confusion (C), characterized by a sense of disturbance and linked to the organization-disorganization dimension, including anxiety and the feeling of cognitive inefficiency.

The adjectives are rated on a 5-point Likert scale (0 = not at all, 1 = a little bit, 2 = moderately, 3 = quite a bit, and 4 = extremely). A Total Mood Disturbance score (TMD) can be calculated by adding the scores for tension, depression, anger, tiredness, and confusion and then subtracting the score for vigor. The POMS showed good reliability both concerning the TMD score (α = 0.85) and the T, D, A, V, S, and C subscales (α = 0.89; α = 0.94; α = 0.71; α = 0.69; α = 0.62; and α = 0.77, respectively).

The SSQ (53) is widely used to measure symptoms of cyber sickness. It comprises 16 items and allows the computation of a total score assessing the severity of the reported symptoms, as well as three subscales for Nausea, Oculomotor Disturbances, and Disorientation (Supplementary Appendix 4).

To measure the attention given to BDZ-related cues, a modified version of the AAS questionnaire (54) will be used, with BDZ-themed questions. Responses are given on a Likert scale ranging from 0 to 10 (Supplementary Appendix 5).

Participants’ sense of presence will be assessed with the PQ (55), comprising 24 items rated on a 7-point Likert scale (Supplementary Appendix 6).

## Development and creation of the virtual environments

**FIGURE 1:**
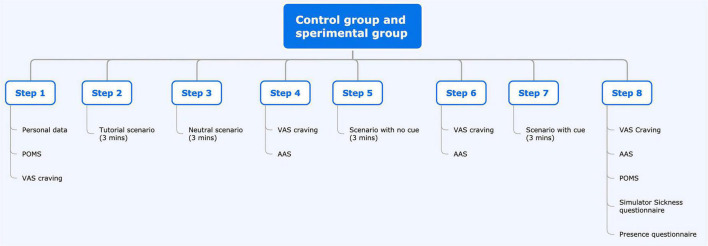
Flowchart.

**FIGURE 2:**
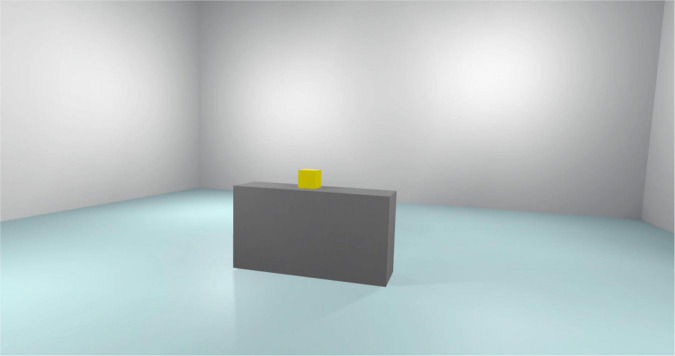
Tutorial scenario.

**FIGURE 3:**
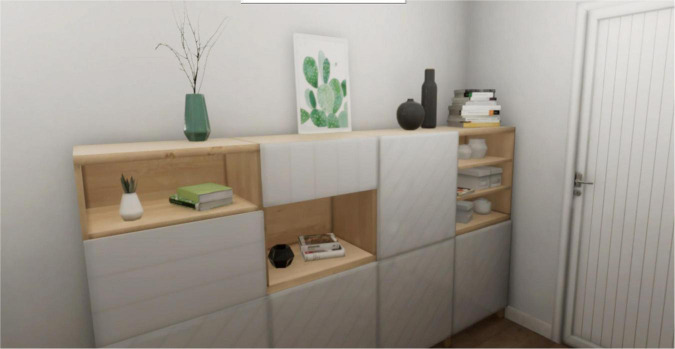
Neutral scenario.

**FIGURE 4:**
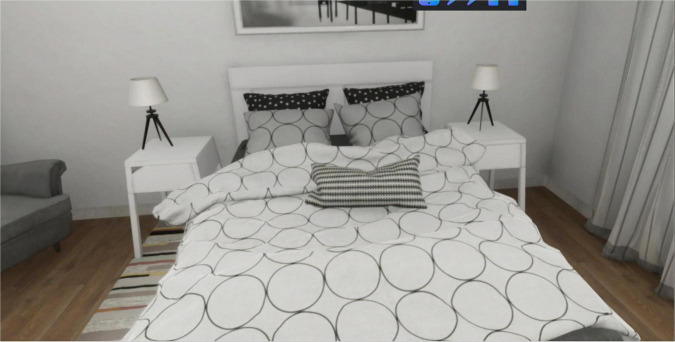
No cue scenario.

**FIGURE 5:**
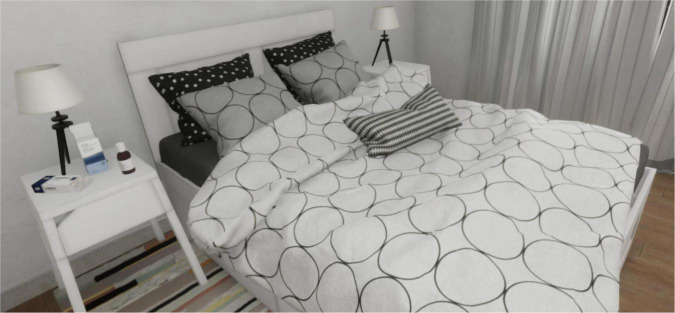
Scenario with cue.

The virtual environments that will be used have been created in photorealistic quality (Figures 1–4) and in “cybersickness-free” mode to allow participants to have a comfortable virtual experience, without any unpleasant side effects. Indeed, cybersickness is a feeling of malaise comprising headaches, vomiting, dizziness, and/or nausea, and it is triggered by a mismatch between visual inputs and those responding to actual movements (56). To achieve this, patients will be allowed to move within the virtual environment through “real” steps whose movement will be faithfully reproduced in the virtual environment. Also, subjects may use teleportation to reach distant positions and beyond the play area. Through the joystick, participants will be able to point to the place they wish to reach, with the virtual experience resuming exactly from the desired spot. The VR environments run on the following VR hardware requirements: (a) HTC-VIVE PRO Full Kit (Figure 5); (b) Gaming PC, Intel Core i7-9700K—GeForce RTX 2070 8GB–16GB DDR4–480GB SSD—Windows 10—Wi-Fi; and (c) a 49“ or 55” TV monitor.

The software has been developed by Hybrid Reality (Padova, Italy^1^), an innovative start-up that developed the scenarios and provides optimization support.

### Virtual environments

Figure 2 reported the tutorial scenario. Participants are exposed to this virtual environment for 3 min. The role of this scenario is to increase familiarity with VR and this is the only scenario in which subjects can interact with the experimenter. In this step of the experiment, the experimenter gives the subjects some instructions and tips on how to better interact with the virtual environment.

Figure 3 reported the neutral scenario. Subjects are exposed to this virtual environment for 3 min. This scenario represents a house entryway. In this scenario, the subject can move freely in the virtual environment, but there are no interactive objects. Every interaction between the subjects and the experimenter is forbidden.

Figure 4 reported the No Cue scenario. Subjects are exposed to this virtual environment for 3 min. This scenario represents a bedroom.

In this scenario, the subject can move freely in the virtual environment, but there are no interactive objects. Every interaction between the subjects and the experimenter is forbidden.

Figure 5 reported the Cue scenario. Subjects are exposed to this virtual environment for 3 min. This scenario represents the same bedroom as the No Cue Scenario. In this scenario, the subject can move freely in the virtual environment. There are only BDZ-related interactive objects. Every interaction between the subjects and the experimenter is forbidden.

**FIGURE 6:**
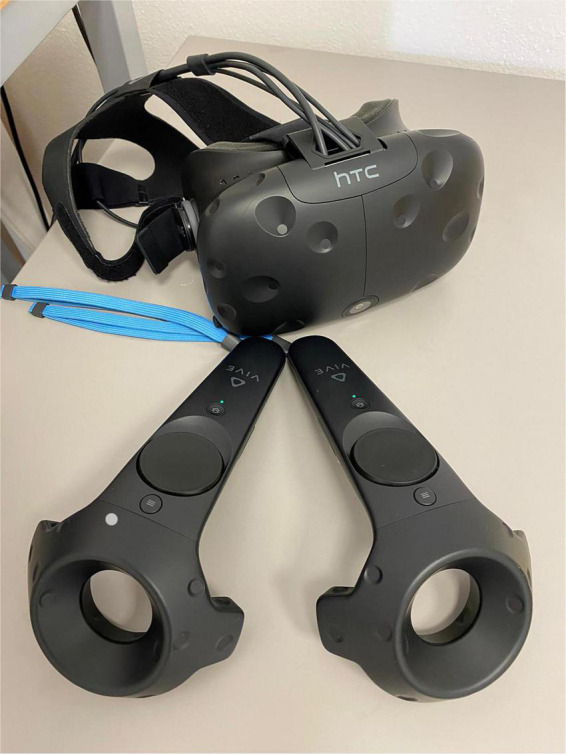
HTC vive.

**FIGURE 7:**
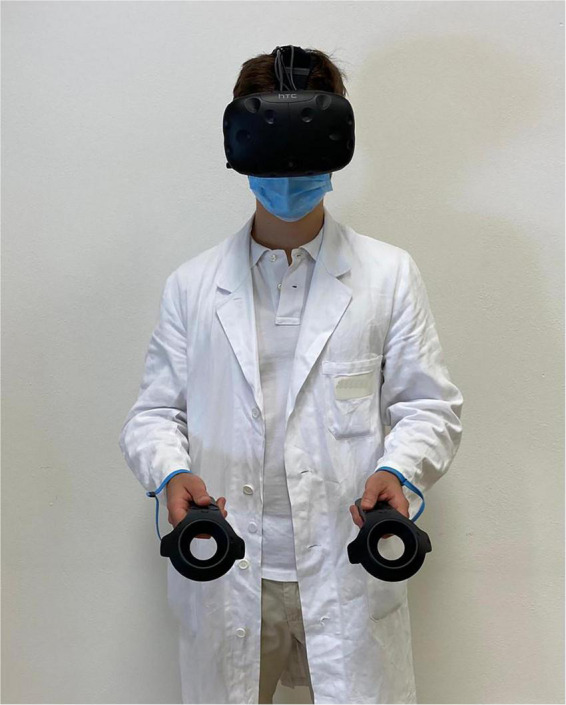
An example of a subject wearing the virtual reality equipment.

Given the COVID-19 pandemic, appropriate accessories will also be employed: disposable, non-woven, breathable face masks for HTC-VIVE PRO, waterproof and hygienic replaceable foam rubber for HTC-VIVE, sanitizing spray, hand sanitizer gel, and surgical masks will be used to guarantee appropriate hygiene of the instruments and the patient’s safety (Figures 6, 7).

### Participants

During the recruitment phase, subjects will be given clear and easy-to-understand information regarding the rationale and purpose of the study, as well as information concerning the possible consequences related to their participation in the study. We will give out informative pamphlets, with detailed information about the research as well as the informed consent, and the subjects will be required to carefully read, fill out, and sign. Both documents will be written in simple language and the name of the person who gave the information to the patient will be listed. The experimenter will further need to sign the informed consent and write the date to validate the document. All procedures will be carried out in accordance with the Declaration of Helsinki.

#### Inclusion criteria

Experimental group:

1. Males and females aged 18–65 years.
2. Subjects with BDZ-use disorder asked to be treated at the Addiction Medicine Unit due to their inability to autonomously quit using BDZs.
3. High-dose BDZ abusers. Although the definition of what constitutes a “high dose” is still controversial and no real consensus exists about the appropriate clinical criteria necessary to define it, we will consider a patient a high-dose user if their BDZ intake will be at least five times higher than the maximum daily defined dose (DDD)

Control group:

1. Males and females aged 18–65 years.
2. Subjects without SUDs (including a BDZ-use disorder) according to ICD 10 F10-F19.

#### Exclusion criteria

At least one of the following:

1. A history of epilepsy or having a first-degree relative with a history of epilepsy.
2. Serious chronic or cardiovascular diseases.
3. Being pregnant.
4. Having a pacemaker or other metal devices on the head and neck, with the exception of piercings and dental braces.
5. Taking psychoactive substances which may interfere with the results of the study.

The presence or absence of each criterion will be assessed by the experimenter before the study begins.

## Safety and hygiene measures

To ensure participant safety and hygiene in the experimental setting, we will comply with the guidelines provided by the Italian Superior Institute of Health (Istituto Superiore di Sanità, ISS), which were approved and implemented in the study by Giordano et al. (57). These include the following procedures:

1. Cleaning of the hands. All staff that will handle the VR devices must use an alcohol-based hand sanitizer. Before a user touches a device, they must wash their hands for at least 40 s, following a specific sequence as illustrated on the Ministry of Health’s website; also, they must rub their hands with an alcohol-based cleaning gel for at least 20 s. This will be done for both operators and participants.
2. The protective waterproof foam guards must be in place on the visor to ensure sanitization of the device.
3. Inserting the waterproof single-use masks in the device before use and substituting them for each subject or operator.
4. Disinfecting all objects that the participants and operators may touch.
5. Surgical masks will be mandatory at all times.
6. Each hygiene measure must be repeated between one participant and the next. At the end of each daily session, all procedures must be enacted one last time to ensure the correct sanitization of the devices.
7. Hospital cleaning personnel will thoroughly clean the room and hospital environment.
8. Trisept Complex, which is a sanitizing product that is available at the internal pharmacy of the University Hospital, will be used to disinfect the VR devices.

## Statistical analyses

### Primary endpoint

To evaluate the association between environmental features and craving, we will use the VAS scale (10 levels) in the experimental group after exposure to the three scenarios: neutral, bedroom without BDZ bottles, and bedroom with bottles similar to the ones containing BDZs.

### Secondary endpoints

To evaluate the association between BDZ craving and mood, affective state, attention, sense of presence, and VR-induced sickness, we will use the total scores and subscales (if present) of the following questionnaires: VAS, POMS, AAS, SSQ, and PQ as measured at the specific timepoints (see flow chart) in the experimental group. The temporal course of the scores will also be compared between the two groups.

### Sample size

The appropriate sample size for this study was computed with the software G*Power 3.1.5.1 (58). We chose to base the computation on the difference between the mean VAS craving scores measured within the subjects after the neutral scenario vs. the BDZ-related scenario. Since we expect this difference to be medium-large, we chose a 0.7 effect size (59). Alpha was set to 0.017 considering multiple comparisons among the three scenarios. Since it will be a pilot study, we choose a two-tailed test with 80% power. The resulting sample size was 25 subjects.

To test if the VR scenarios can appropriately distinguish between BDZ abusers and controls, we will also recruit 25 healthy subjects, bringing the total sample size to 50.

## Data analysis

All the variables considered in the study will be analyzed by using their most appropriate descriptive statistic. In particular, we will use mean and standard deviation for normally distributed continuous variables, median and interquartile range for non-normally distributed variables, and frequency distribution for categorical variables.

In the experimental group, we will perform a one-way ANOVA for VAS craving at each study timepoint, meaning after exposure to each scenario.

After the repeated-measures ANOVA, we will conduct Bonferroni’s multiple comparison correction to compute *post-hoc* comparisons and test significant contrasts among the various timepoints for VAS craving. Should the ANOVA assumptions be violated, a Friedman test will be performed. In the experimental group, we will also compute Pearson’s correlations or Spearman’s rank coefficients among VAS, POMS, AAS, SSQ, and PQ scores measured at the same timepoints. We will also explore the correlations between VAS score variations in the three scenarios (Δ VAS neutral—bedroom without BDZ; Δ VAS neutral—bedroom with BDZs; Δ VAS bedroom without BDZs—bedroom with BDZs) and the POMS, AAS, SSQ, and PQ score variations (Δ).

Finally, we will use multilevel linear models to assess group differences in the VAS, POMS, AAS, SQ, and PQ scores as repeatedly measured in the same subjects at specific timepoints. All statistical analyses will be conducted using the PRISM6 software (GraphPad, CA, USA).

## Study plan

Flow chart of the study for the experimental and control groups.

### Ethics statement

Approval for the research was obtained from the Ethics Committee for Clinical Trials (CESC) of the Provinces of Verona and Rovigo based at the Integrated University Hospital of Verona, Italy (approval code: 3624CESC with Protocol No. 16883 of 09-03-2022). The latest revision of the Declaration of Helsinki as well as the Oviedo Declaration is the basis for the ethical conduct of the study. The study protocol is designed and will be conducted to ensure adherence to the principles and procedures of Good Clinical Practice and to comply with Italian law, as described in the following documents and accepted, by signature, by the study investigators: ICH Harmonized Tripartite Guidelines for Good Clinical Practice 1996; Directive 91/507/EEC, The Rules Governing Medicinal Products in the European Community; D. L.vo n. 211 of 24 June 2003; D. L.vo n. 200, 6 November 2007; Ministerial Decree of 21 December 2007; AIFA Determination, 20 March 2008. All essential clinical records will be retained to demonstrate the validity of the study and the integrity of the data collected. The promoter of this study, in accordance with the responsibilities required by the rules of good clinical practice (Legislative Decree 211/2003) and in accordance with the laws and regulations regarding data protection (including the European Regulation on the protection of personal data 2016/679), will process the personal data that will be collected exclusively for the implementation of the study and for device surveillance.

## Discussion

Benzodiazepines are among the most widely used psychotropic medications worldwide, but the chronic use of BDZs can cause several deficits. The risk of dependence after long-term use has been widely reported, and abrupt withdrawal of the drug causes several unpleasant symptoms. In addition to subjects that begin using BDZs to treat anxiety and insomnia and end up using them inappropriately, some subjects deliberately abuse BDZs. In this case, BDZs are taken to counter anxiety or to enhance the effects of other drugs, such as alcohol or opioids, in what becomes a polydrug use pattern (15, 60). Withdrawal syndrome, even from therapeutic doses of BDZs, can be severe and, in some cases, may preclude the patient from ceasing the use of the drug (32, 61). Notwithstanding the important presence of BDZs in clinical practice, no studies have analyzed CR in the context of BDZ addiction yet. VR is a promising research tool since it creates a state of immersion closer to reality, but that still allows the measure of neuropsychological and behavioral responses in a more controlled way (62). For this reason, VR has been extensively used in addiction to drugs and tobacco, for example, to explore smoking withdrawal, craving, and cue reactivity (62). These VR reports, while confirming the findings that were demonstrated in previous, traditional laboratory studies (i.e., cues presented as pictures or videos), still need to better characterize VR-triggered cue reactivity. Environmentally induced craving has been described for various SUDs, and especially for alcohol and tobacco, in which the subjects who were exposed to abuse-related VR stimuli manifested increased craving (47, 63, 64). This has not been investigated in BDZ dependence, but looking at literature concerning other substances, we expect that CR may also be involved in BDZ addiction. Therefore, we expect increased craving in BDZ abusers exposed to BDZ-like stimuli in VR settings, and also significant differences in craving between the experimental group and the control group, with the former exhibiting higher levels of overall craving. One of the most critical issues regard the design of complex and personalized experimental sessions that would also allow measuring and standardizing the variables and parameters of interest (65).

## Conclusion

Studies on BDZ abuse are not many, especially concerning high-dose abusers. There are still many relatively unknown variables that would nevertheless be important to investigate, such as craving.

BDZ craving is clinically characterized as an incontrollable urge to take the target substance when it is not readily available. Unlike what has been done for alcohol and tobacco, environmentally induced craving is often considered absent in BDZ abuse and, therefore, it has not yet been investigated in BDZ users. Its role in BDZ abuse, however, has never been tested in scientific research, and neither has its actual presence or absence in this SUD. Virtual reality, therefore, enables the study of this phenomenon without exposing the subjects to real risks. This study protocol aims to fill the current gaps in the scientific literature concerning BDZ-evoked craving in high-dose BDZ users and to better characterize the possible role of the environment in this important mechanism.

## Ethics statement

The studies involving human participants were reviewed and approved by Ethics Committee for Clinical Trials (CESC) of the Provinces of Verona and Rovigo based at the Integrated University Hospital of Verona, Italy (approval code: 3624CESC with Protocol No. 16883 of 09-03-2022). The patients/participants provided their written informed consent to participate in this study.

## Author contributions

LZ, CC, and FL: conceptualization and writing review and editing. LZ: data curation and investigation. GV: statistical analysis. CC and LZ: methodology. FL and CC: supervision. SC, FF, ST, AC, VM, MM, and VS: writing original draft. All authors have read and agreed to the published version of the manuscript.

## Footnotes

1
https://www.hybridreality.it
