# Supplementary material for: Study protocol—Evoked craving in high-dose benzodiazepine users
Source: Front Psychiatry. 2022 Oct 13;13:956892. doi: 10.3389/fpsyt.2022.956892 (PMC9608779; doi:10.3389/fpsyt.2022.956892)
Supplement: Supplementary file 1 [file Table_1.DOC]

**Appendix 1**

**Studio sul Craving evocato in pazienti assuntori di benzodiazepine**

**(VR_BDZ-2020)**

CASE REPORT FORM

**QUESTIONARIO DEMOGRAFICO**

| **CODICE SOGGETTO** |  |
| --- | --- |
| **DATA DI RECLUTAMENTO** | ___/___/_____ |
| **SESSO** | - M - F |
| **ETA’** | _______ anni |
| **GRUPPO DI APPARTENENZA** | - Gruppo di controllo - Gruppo sperimentale |
| **CRITERI DI INCLUSIONE**  ***(Il soggetto deve presentare le caratteristiche riportate a destra)*** | - Età compresa tra 18 - 65 anni - Soggetto affetti da disturbo da uso di BDZ (solo per gr. sperimentale) - Soggetto che non presenta disturbo da uso di sostanze (tra cui BDZ) – ICD10 F10-F19 (solo per gr. di controllo) |
| **CRITERI DI ESCLUSIONE**  ***(Se il soggetto presenta uno dei criteri riportati a destra, non deve essere reclutato)*** | - Anamnesi positiva per epilessia o familiari di primo grado con storia clinica di epilessia; - Anamnesi positiva per malattie cardiovascolari o croniche gravi; - Gravidanza; - Presenza di pacemaker cardiaco o di altri dispositivi metallici nel distretto testa – collo, ad eccezione di piercing o apparecchi ortodontici; - Assunzione di sostanze psicoattive che potrebbero interferire con i risultati dello studio |
| **INFORMAZIONI SU DIPENDENZA DA SOSTANZE PSICOATTIVE** | Assumi sostanze psicoattive (incluse BDZ)?   - SI - NO   Se sì, quali? __________________________________ |
| **INFORMAZIONI SU DIPENDENZA DA BDZ *(solo per il gruppo sperimentale)*** | Che tipologia di benzodiazepina assumi?  ______________________________________  Dosaggio assunto di BDZ:  _______________________________________  Da quanto tempo assumi BDZ?  ________________________________________ |
| **METERIALE DA CONSEGNARE AL PARTECIPANTE** | - Nota informativa ed espressione del consenso informato - Consenso al trattamento dei dati personali - Lettera informativa per il medico curante |
