# Supplementary material for: Study protocol—Evoked craving in high-dose benzodiazepine users
Source: Front Psychiatry. 2022 Oct 13;13:956892. doi: 10.3389/fpsyt.2022.956892 (PMC9608779; doi:10.3389/fpsyt.2022.956892)

**Appendix 2**

**QUESTIONARI T_0_:**

**Da sottoporre al partecipante prima di iniziare la sessione di VR**

1. **SCALA VAS**

Quanta voglia hai di assumere una benzodiazepina in questo momento?


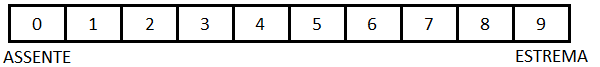

Supplement: Supplementary file 2 [file Table_2.DOC]
