# Supplementary material for: Study protocol—Evoked craving in high-dose benzodiazepine users
Source: Front Psychiatry. 2022 Oct 13;13:956892. doi: 10.3389/fpsyt.2022.956892 (PMC9608779; doi:10.3389/fpsyt.2022.956892)
Supplement: Supplementary file 3 [file Table_3.DOC]

**Appendix 3**

**POMS**

La seguente lista comprende sensazioni che tutti possiamo avere. Per favore legga con attenzione ciascuna voce e scelga il punteggio che meglio descrive come si sente in questo momento.

|  | Per nulla  **0** | Poco  **1** | Abbastanza  **2** | Molto  **3** | Moltissimo  **4** |
| --- | --- | --- | --- | --- | --- |
|  |
| 1. Teso |  |  |  |  |  |
| 1. Arrabbiato |  |  |  |  |  |
| 1. Stressato |  |  |  |  |  |
| 1. Infelice |  |  |  |  |  |
| 1. Pieno di vita |  |  |  |  |  |
| 1. Con le idee confuse |  |  |  |  |  |
| 1. Scontento di quello che ho fatto |  |  |  |  |  |
| 1. Con un tremito diffuso |  |  |  |  |  |
| 1. Svogliato |  |  |  |  |  |
| 1. Irritato |  |  |  |  |  |
| 1. Malinconico |  |  |  |  |  |
| 1. Attivo |  |  |  |  |  |
| 1. Coi nervi a fior di pelle |  |  |  |  |  |
| 1. Immusonito |  |  |  |  |  |
| 1. Triste |  |  |  |  |  |
| 1. Pieno di energia |  |  |  |  |  |
| 1. In preda al panico |  |  |  |  |  |
| 1. Senza speranza |  |  |  |  |  |
| 1. Rilassato |  |  |  |  |  |
| 1. Degno di disprezzo |  |  |  |  |  |
| 1. Scontroso |  |  |  |  |  |
| 1. A disagio |  |  |  |  |  |
| 1. Irrequieto |  |  |  |  |  |
| 1. Incapace di concentrarmi |  |  |  |  |  |
| 1. Stanco |  |  |  |  |  |
| 1. Seccato |  |  |  |  |  |
| 1. Scoraggiato |  |  |  |  |  |
| 1. Pieno di risentimento |  |  |  |  |  |
| 1. Nervoso |  |  |  |  |  |
| 1. Solo, isolato dagli altri |  |  |  |  |  |
| 1. Avvilito |  |  |  |  |  |
| 1. Scombussolato |  |  |  |  |  |
| 1. Di buon umore |  |  |  |  |  |
| 1. Amareggiato |  |  |  |  |  |
| 1. Esaurito |  |  |  |  |  |
| 1. Ansioso |  |  |  |  |  |
| 1. Pronto ad attaccar briga |  |  |  |  |  |
| 1. Di umore nero |  |  |  |  |  |
| 1. Sfiduciato |  |  |  |  |  |
| 1. Indolente |  |  |  |  |  |
| 1. Ribelle |  |  |  |  |  |
| 1. Senza aiuto, abbandonato |  |  |  |  |  |
| 1. Annoiato |  |  |  |  |  |
| 1. Perplesso |  |  |  |  |  |
| 1. Pronto, in gamba |  |  |  |  |  |
| 1. Deluso |  |  |  |  |  |
| 1. Furibondo |  |  |  |  |  |
| 1. Efficiente |  |  |  |  |  |
| 1. Pieno di iniziativa |  |  |  |  |  |
| 1. Di cattivo umore |  |  |  |  |  |
| 1. Persona di poco valore |  |  |  |  |  |
| 1. Smemorato |  |  |  |  |  |
| 1. Libero da preoccupazioni |  |  |  |  |  |
| 1. Terrorizzato |  |  |  |  |  |
| 1. Tormentato dai rimorsi |  |  |  |  |  |
| 1. Forte |  |  |  |  |  |
| 1. Indeciso su cosa fare |  |  |  |  |  |
| 1. Frastornato |  |  |  |  |  |
