# Supplementary material for: Study protocol—Evoked craving in high-dose benzodiazepine users
Source: Front Psychiatry. 2022 Oct 13;13:956892. doi: 10.3389/fpsyt.2022.956892 (PMC9608779; doi:10.3389/fpsyt.2022.956892)
Supplement: Supplementary file 4 [file Table_4.DOC]

**Appendix 4**

**SIMULATOR SICKNESS QUESTIONNAIRE**

SIMULATOR SICKNESS QUESTIONNAIRE (SSQ) - Kennedy, Lane, Berbaum, & Lilienthal (1993)

Istruzioni: cerchiare l’aggettivo che definisce l’intensità della sensazione che avverte in questo momento

.

| **1.Malessere generale** | Nessuno | Lieve | Moderato | Severo |
| --- | --- | --- | --- | --- |
| **2.Affaticamento** | Nessuno | Lieve | Moderato | Severo |
| **3.Mal di testa** | Nessuno | Lieve | Moderato | Severo |
| **4.Affaticamento oculare** | Nessuno | Lieve | Moderato | Severo |
| **5.Difficoltà nel mettere a fuoco le immagini** | Nessuno | Lieve | Moderato | Severo |
| **6.Incremento della salivazione** | Nessuno | Lieve | Moderato | Severo |
| **7.Sudorazione** | Nessuno | Lieve | Moderato | Severo |
| **8.Nausea** | Nessuno | Lieve | Moderato | Severo |
| **9.Difficoltà di concentrazione** | Nessuno | Lieve | Moderato | Severo |
| **10.Sensazione di pesantezza alla testa** | Nessuno | Lieve | Moderato | Severo |
| **11.Visione offuscata** | Nessuno | Lieve | Moderato | Severo |
| **12.Sensazione di stordimento con gli occhi aperti** | Nessuno | Lieve | Moderato | Severo |
| **13.Sensazione di stordimento con gli occhi chiusi** | Nessuno | Lieve | Moderato | Severo |
| **14.Vertigine *** | Nessuno | Lieve | Moderato | Severo |
| **15. Sensazione di stomaco **** | Nessuno | Lieve | Moderato | Severo |
| **16.Eruttazione** | Nessuno | Lieve | Moderato | Severo |

*La vertigine è avvertita come una perdita di orientamento correlata alla posizione eretta

** Il termine sensazione di stomaco è solitamente usato per indicare una sensazione di fastidio allo stomaco vicina alla nausea.

Ultima versione: Marzo 2013

*** Verisone originale: Kennedy, R.S., Lane, N.E., Berbaum, K.S., & Lilienthal, M.G. (1993).

Simulator Sickness Questionnaire: An enhanced method for quantifying simulator sickness. International Journal of Aviation Psychology, 3(3), 203-220

1. **QUESTIONARIO SUL SENSO DI PRESENZA (PRESENCE QUESTIONNAIRE, PQ)** (Witmer & Singer, versione 3.0, novembre 1994)* Revisionato dal Laboratorio di Cybertecnologia UQO (2004)
