# Supplementary material for: Study protocol—Evoked craving in high-dose benzodiazepine users
Source: Front Psychiatry. 2022 Oct 13;13:956892. doi: 10.3389/fpsyt.2022.956892 (PMC9608779; doi:10.3389/fpsyt.2022.956892)
Supplement: Supplementary file 5 [file Table_5.DOC]

**Appendix 6**

**QUESTIONARIO SUL SENSO DI PRESENZA (PRESENCE QUESTIONNAIRE, PQ)** (Witmer & Singer, versione 3.0, novembre 1994)* Revisionato al Laboratorio di Cybertecnologia UQO (2004)
