# Supplementary material for: Study protocol—Evoked craving in high-dose benzodiazepine users
Source: Front Psychiatry. 2022 Oct 13;13:956892. doi: 10.3389/fpsyt.2022.956892 (PMC9608779; doi:10.3389/fpsyt.2022.956892)
Supplement: Supplementary file 6 [file Table_6.DOC]

**Appendix 5**

**SCALA AAS:** A modified version of the Alcohol Attention Scale (AAS) (Monti et al., 1993)

1- Quanta attenzione hai prestato alla vista di benzodiazepine nella stanza?

| 0 Non mi sono accorto | 1 | 2 | 3 | 5 | 6 | 7 | 8 | 9 | 10 ho prestato una completa attenzione |
| --- | --- | --- | --- | --- | --- | --- | --- | --- | --- |

2- Quanto hai pensato di assumere benzodiazepine mentre eri nella stanza?

| 0 Non pensavo affatto ad assumere benzodiazepine | 1 | 2 | 3 | 5 | 6 | 7 | 8 | 9 | 10 pensavo ad assumere benzodiazepine tutto il tempo |
| --- | --- | --- | --- | --- | --- | --- | --- | --- | --- |
